# Tempering the Untempered: Reflections on Growth and Countertransference

**DOI:** 10.1007/s40596-025-02290-9

**Published:** 2025-12-18

**Authors:** H. Yavuz Ince

**Affiliations:** Department of Psychiatry, Division of Child and Adolescent Psychiatry, University of Michigan, Ann Arbor, MI USA

After a long and tiring clinic day at outpatient child and adolescent psychiatry, I start co-signing trainees’ notes. One sentence toward the end of a note draws my attention: “Of note, throughout the appointment, mom was overelaborative, constantly made demands, was irritable at times.” I search my memory to make sure I remember correctly, but I don’t recall the mother being unreasonable or demanding. I think she was appropriately concerned and wanted to understand how we conceptualized her child’s difficulties. I feel baffled for two reasons. First, I couldn’t believe that my trainee and I, both mental health professionals, had such different perceptions of a shared experience. Second, I question my own teaching skills for not creating a space where my trainee could vent and debrief.

I am by no means the most self-aware person in the world. Despite being a psychiatrist, I sometimes miss subtle nonverbals. I sometimes say awkward things and feel bad afterward. Like any psychiatrist, I have “mental patient encounter recordings” that I “replay” from time to time, the moments that make me cringe but help me make sure I don’t repeat the same mistake. I know I am human. I also know that trainees are human. Yet I wonder how this skill can be taught without shame or guilt.

Gabbard reminds us that countertransference is not merely a reaction to the patient but a mirror into the clinician’s inner world, a tool for understanding rather than judgment [1]. Every psychiatry trainee knows what countertransference means, but how can they notice it in the moment? And for us, as supervisors, how do we correct them tactfully without wounding and making trainees feel vulnerable? How can we help them grow to be appropriately boundaried without making them perceived by their patients as cold, insensitive, or even worse, indifferent?

## Trainee’s World

There are trainee-related factors: their level of psychological mindedness, level of curiosity, and openness. Trainees, like all human beings, often carry invisible burdens such as caregiving responsibilities, relationship struggles, self-doubt, and many others. They live in a world where immediacy is expected: rapid replies to long and complex portal messages, rapid solutions to long-standing problems. They function in a society where speed is rewarded more than reflection. In this climate, boundaries are no longer just deontologically necessary but essential for preserving their sanity and for preventing burnout. Most importantly, they are expected to show up every single day in a rapidly growing and expanding profession they are still learning, as high-achieving, conscientious individuals. They face with daily vulnerability that threatens the very self-esteem that they need to move forward in their profession.

## Supervisor’s Task

Supervisors must be present and lead by example. They must scaffold, correct without shame, and be neutral, descriptive, and sensitive. True learning only occurs when the trainee feels safe. How we maintain that safety without infantilizing the trainee depends on the situation and the individual. Over the years, I have found that the old ethical maxim, “treat others the way you want to be treated,” remains the best litmus test. I often non-judgmentally and quite curiously ask trainees how they would wish to be treated if they were in their patient’s position. Creating such reflective spaces not only teaches empathy but also acknowledges what Meier and colleagues describe as the “inner life” of physicians, which encompasses the emotional core that shapes how we care for both others and ourselves [2].

For this to work, supervisors need to know a little about the trainee. If the trainee has dogs, for example, I might ask them to recall their experience at the vet, when the vet spoke quickly or used terms that were hard to understand for the general public. How did they feel in that moment? Despite being a medical school graduate, did they find it hard to follow the recommendations?

Knowing enough about the trainee to understand possible routes of countertransference helps supervisors act proactively. Program directors and other leaders should also inform teaching faculty about unique factors that may impact this transfer. Supervising faculty should also have enough time to discuss patients and reflect on cases that they staff.

## Finding the Balance

I never imagined reaching a point in my career where I would say, “Back in my time.” Yet, it has become clear that the expectations between trainees and supervisors evolve with the zeitgeist [3]. Educators and learners must continually examine and discuss new social norms in medical education to ensure that the unwritten rules we accept actually serve both the trainees and the community.

Supervisors have to be brave to challenge their trainees without shaming and wounding them. As Rumi once said, “The wound is the place where the light enters you.” I would add, as a student of biology, that sometimes wounds heal with scars. Those scars are made of fibrous tissue, and they can make the light’s passage a little harder. Growth is a process of constant calibration, often helping trainees recognize where their wounds have scarred. In doing so, they can learn when to soften (which may be more difficult for wounded tissue) and when to hold firm. The challenge and beauty of teaching lie in rediscovering that balance each day within oneself and within others.

## Data Availability

No datasets were generated or analyzed during the current study.
